# Tuning Gaps and Schottky Contacts of Graphene/Phosphorene Heterostructures by Vertical Electric Field and Strain

**DOI:** 10.3390/nano13162358

**Published:** 2023-08-17

**Authors:** Alessia Muroni, Simone Brozzesi, Friedhelm Bechstedt, Paola Gori, Olivia Pulci

**Affiliations:** 1 Department of Physics, University of Rome ‘Tor Vergata’ and INFN, Via della Ricerca Scientifica 1, 00133 Rome, Italy; alessia.muroni@roma2.infn.it (A.M.); simone.brozzesi@roma2.infn.it (S.B.); olivia.pulci@roma2.infn.it (O.P.); 2 Institut für Festkörpertheorie und -Optik, Friedrich-Schiller-Universität Jena, Max-Wien-Platz 1, 07743 Jena, Germany; 3 Department of Industrial, Electronic and Mechanical Engineering, Roma Tre University, Via della Vasca Navale 79, 00146 Rome, Italy

**Keywords:** 2D materials, graphene, phosphorene, vdW heterostructure, Schottky contact, band structure, gap, strain, electric field

## Abstract

We present a comprehensive study of the structural and electronic properties of a graphene/phosphorene (G/P) heterostructure in the framework of density functional theory, including van der Waals interaction in the exchange–correlation functional. While the G(4 × 1)/P(3 × 1) superlattice usually used in the literature is subject to a strain as high as about 7%, the in-plane strain could be drastically reduced to under 1% in the G(4 × 13)/P(3 × 12) heterostructure investigated here. Adapting the lattice constants of the rectangular lattices, the equilibrium configuration in the *xy* plane of phosphorene relative to the graphene layer is optimized. This results in an equilibrium interlayer distance of 3.5 Å and a binding energy per carbon atom of 37 meV, confirming the presence of weak van der Waals interaction between the graphene and the phosphorene layers. The electronic properties of the heterostructure are evaluated under different values of interlayer distance, strain and applied vertical electric field. We demonstrate that G/P heterostructures form an *n*-type Schottky contact, which can be transformed into *p*-type under external perturbations. These findings, together with the possibility to control the gaps and barrier heights, suggest that G/P heterostructures are promising for novel applications in electronics and may open a new avenue for the realization of innovative optoelectronic devices.

## 1. Introduction

Graphite crystals are a possible source to exfoliate single atomic layers of carbon atoms, namely graphene (G) [1,2]. Great efforts have been devoted over the years to find similarly structure elemental crystals which also allow for mechanical exfoliation of true 2D materials [3,4]. Among them, the most stable as well as reactive form of elemental phosphorus is black phosphorus (BP), a narrow-gap semiconductor with a direct gap of about 0.3 eV [4]. Bulk phosphorus exists in several allotropic forms; the orthorombic one is the most stable polymorph under ambient conditions, and it has a puckered layer structure. As isolated object such a BP layer, also known as phosphorene, has outstanding mechanical, thermal and optical properties due to puckering, as well as significant in-plane anisotropy and low point-group symmetry C${}_{2h}$ [5,6].

Phosphorene shows several unique properties: it possesses a peculiar band structure whose dispersion is nearly linear along the armchair directions but parabolic in the perpendicular directions [5,7]. Depending on the number of phosphorene layers, it is possible to find a tunable band gap between 0.3 eV (bulk or five layers) and 2.0 eV (isolated layer) [8,9,10]. As a paradigm, one identifies two types of Dirac fermions in the low-energy spectrum. One pair of type I Dirac points sits on high-symmetry lines, while two pairs of type II Dirac points are located at generic k-points [11]. In BP nanoribbons, quasi-flat edge bands appear, and their emergence can be explained by topological arguments [12]. The application of a vertical electric field can switch the system character from topological to trivial insulator [13], similar to the case of silicene, germanene or stanene [14]. Band-gap engineering with in-plane or out-of-plane strain may result in direct–indirect semiconductor and metal–insulator transitions [5,15,16]. An extraordinary phenomenon is the observation of excitons with large binding energies, which generally appear in 2D semiconductors because of reduced screening [17]; moreover, strongly layer-dependent exciton binding energies up to slightly below 1 eV have been observed for phosphorene monolayers [18,19,20,21,22,23,24]. Other remarkable anisotropic properties concern the thermal transport [25] and the polarization-dependent optical spectra [24,26].

Phosphorene or, more generally, few-layer BP, possesses not only anisotropic but also very high carrier mobilities [9,27], with the high hole mobility having a peak field value of about 1000 cm${}^{2}$/Vs [4,28], which is why few-layer phosphorene can be applied as an emerging electrode material for electrochemical energy storage technologies [29]. In fact, in combination with other materials, e.g., graphene, the resulting heterostructure may be applicable as anode material for rechargeable lithium-ion batteries [30]. Moreover, BP is the only elemental 2D material apart from graphene [31] that is capable of supporting electronic devices [4], allowing for the fabrication of field-effect transistors (FETs) with an on/off current ratio up to 10${}^{5}$ at room temperature [4,9]. Phosphorene is predicted to be one of the most promising 2D channel materials for high-speed and flexible radio-frequency nanosystems [32,33]. In such devices, the interface of phosphorene and the electrodes plays a fundamental role. For instance, metallic nickel (Ni) with a large work function reduces the Schottky barrier height $\Phi_{B}$ in a BP FET after thermal annealing due to the formation of Ni–P alloy [34]. As an alternative to nickel or gold, graphene might prove advantageous as a contact material for obtaining low-cost, highly flexible and small-size devices. Such devices may consist of a phosphorene channel protected by graphene sheets working as contacts and may be divided into source and drain by local hydrogenation of graphene towards insulating graphane [35]. The appearance of van der Waals (vdW)-bonded graphene/phosphorene (G/P) heterostructures give rise to Schottky barriers for electrons (or holes), which should be strongly influenced by normal electric fields or in-plane/out-of-plane strains [36,37,38]. For sure, BP is a versatile material suitable for numerous applications; it could be employed in the production of transistors and photovoltaic devices [39], or it could be used as a potential anode for magnesium- or lithium-ion batteries [40,41].

However, phosphorene presents different limitations concerning its application in electronic devices, since it is unstable under environmental conditions [42]. To overcome this problem, BP is often combined with other materials capable of forming stable heterostructures, such as G/P. A strongly strained vdW heterostructure forms a *p*-type Schottky contact at the G/P interface, which could be transformed into an *n*-type Schottky contact varying, for example, the interlayer distance [43], the imposed strain on graphene and phosphorene layers [36] or the applied electric field perpendicular to the layers [44]. The influence on electron transport of the twist angle has been also investigated [45].

In this work, we investigate the structural and electronic properties of crystalline G/P heterostructures as a function of the interlayer distance and of an applied vertical electric field. We use a large coincidence lattice to avoid artificial in-plane strains due to too small cells used in the modeling shown by most theoretical studies. The main method is the density functional theory (DFT) including vdW interaction in the exchange–correlation (XC) functional. The atomic geometries of the resulting heterostructures are compared with those of the isolated 2D crystals. The accompanying electronic structures are tuned with the G/P layer distance and an external electric field. In particular, the Schottky barrier heights for electrons and holes, as well as their modification, are investigated.

## 2. Model and Computational Methods

We carry out the first-principles simulations within the DFT implemented in the Quantum Espresso suite [46,47]. The generalized gradient approximation of Perdew, Burke and Ernzerhof (GGA-PBE) [48] is utilized to describe the XC functional. The vdW corrections proposed by Grimme (DFT-D2) [49] are included to better describe the long-range interactions. We use a plane-wave cut-off energy of 110 Ry, which guarantees a total energy convergence below 1 meV/atom. All the geometrical structures are fully relaxed until the forces are converged to 10 meV/Å. Calculated lattice parameters for the pristine graphene and rectangular phosphorene are *a*${}_{G}$ = 2.457 Å, *a*${}_{P}$ = 3.307 Å and *b*${}_{P}$ = 4.587 Å, which are in good agreement with theoretical [24,38,50,51] and experimental values (*a*${}_{G}$ = 2.46 Å, *a*${}_{P}$ = 3.35 Å and *b*${}_{P}$ = 4.62 Å) [3,9].

**Figure 1:**
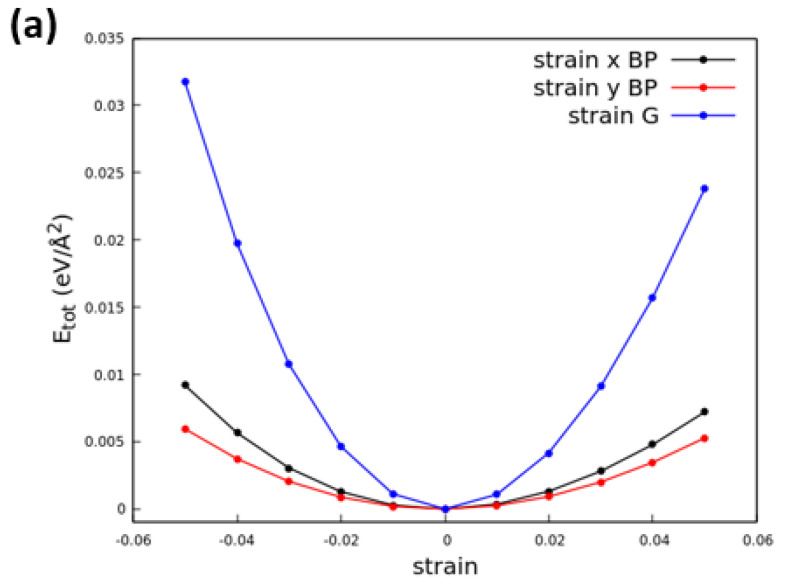

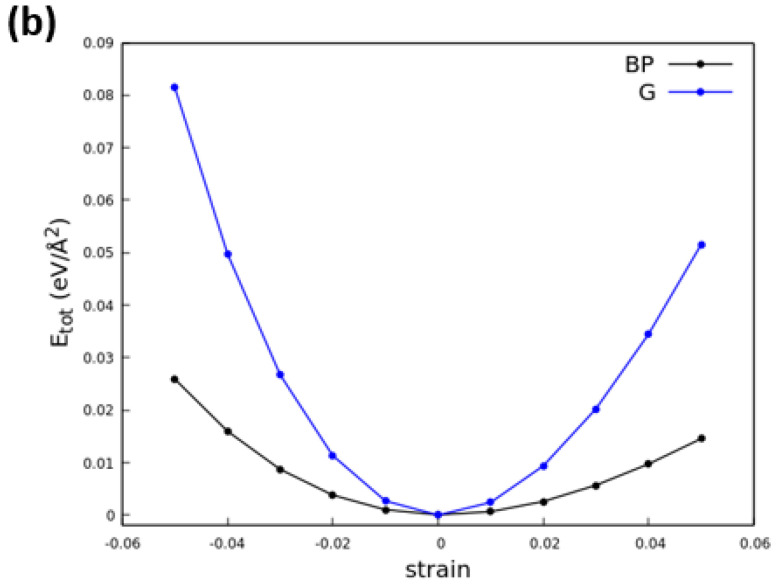
Total energy as a function of lateral uniaxial (**a**) and biaxial (**b**) strain in phosphorene and graphene layers.

The construction of a reasonable coincidence lattice between graphene and phosphorene asks for a representation of graphene using a nonprimitive rectangular unit cell with lattice constants *a*${}_{G}$ = 2.457 Å and *b*${}_{G}$ = 4.257 Å. Since the lattice parameters of an 1 × 1 rectangular graphene unit cell (made of 4 C atoms) are significantly different from those of the 1 × 1 phosphorene unit cell (made of 4 P atoms), the use of an adequate number of cells for the two layers along the *x* and *y* directions is required to obtain a minimal *mismatch* f${}_{x}$ = 2 $\frac{a_{G}-a_{P}}{a_{G}+a_{P}}$ and f${}_{y}$ = 2 $\frac{b_{G}-b_{P}}{b_{G}+b_{P}}$ between graphene and phosphorene lattice parameters. Several research teams studied the heterostructure G/P consisting of a 4 × 1 graphene supercell and a 3 × 1 phosphorene supercell, which has f${}_{y}$ = −0.075 and f${}_{x}$ = −0.009. The resulting tensile uniaxial strain in graphene is small (around 0.5%), but the compressive uniaxial strain in phosphorene is 6.8% along *y*, and it will significantly influence the structural and electronic properties of the G/P heterostructure. This is illustrated in Figure 1 for the total energies. Calculations show that graphene (blue curves) is more rigid than phosphorene (red and black curves), which in addition exhibits a well-known asymmetry between uniaxial strains in the *x* and *y* directions. Significant changes may occur when increasing or decreasing the lattice constants by about 7%. A correspondingly large effect is expected for the band structure and the gap opening in phosphorene and, to a minor extent, for the Fermi velocities of the Dirac bands. Given the presence of an excessively large mismatch along the *y* direction and a huge resulting strain in the G/P heterostructure, we resorted to considering a larger coincidence lattice [52] using a supercell composed of 4 × 13 unit cells of graphene and 3 × 12 unit cells of phosphorene along the *x* and *y* directions. The lattice constants are now $a_{G(4\times 13)}$ = 9.830 Å, *b*${}_{G(4\times 13)}$ = 55.335 Å, *a*${}_{P(3\times 12)}$ = 9.921 Å and *b*${}_{P(3\times 12)}$ = 55.039 Å. As a result, the lattice mismatch between graphene and phosphorene in this larger supercell is reduced (f${}_{x}$ = −0.009 and f${}_{y}$ = 0.005). The in-plane strains result to be $\epsilon_{xx}^{G}$ = 0.98%, $\epsilon_{yy}^{G}$ = −0.16% and $\epsilon_{xx}^{P}$ = −0.81%, $\epsilon_{yy}^{P}$ = 0.38% for graphene and phosphorene, respectively. These strains are less than 1%.

At this stage, to apply the plane-wave expansion implemented in the Quantum Espresso suite, the heterostructures are periodically repeated in normal direction. Therefore, a vacuum space of 15 Å in the *z*-direction is set to avoid spurious interactions between neighboring slabs. The 2D Brillouin zone (BZ) is sampled with a 9×3×1 Monkhorst–Pack *k*-point mesh.

## 3. Results and Discussion

### 3.1. Structure and Energetics

**Figure 2:**
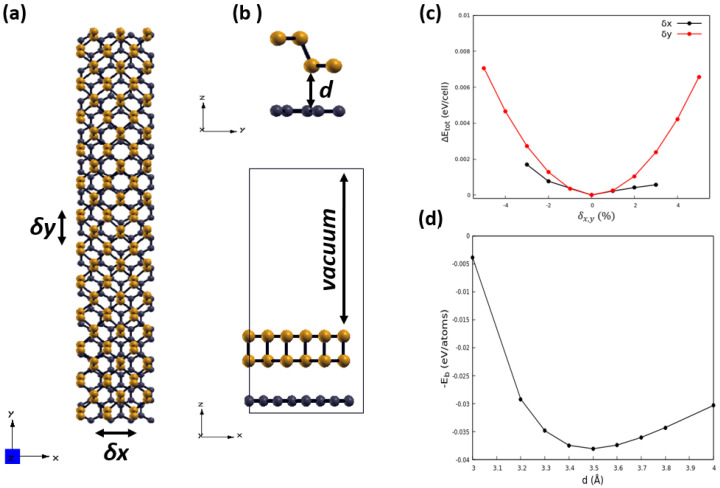
(**a**,**b**) Top view and side view of the G(4 × 13)/P(3 × 12) heterostructure: Black and orange spheres stand for carbon and phosphorus atoms, respectively. The interlayer distance between graphene and phosphorene layers is marked by the letter *d*. (**c**) Evolution of the total energy as a function of displacement $\delta_{x}$ and $\delta_{y}$ of the phosphorene layer relative to graphene. (**d**) Negative binding energy as a function of the interlayer distance.

To obtain the equilibrium configuration, we apply a three-step procedure: (1) due to the much stronger stiffness of the graphene lattice as compared with BP (see Figure 1), we impose as starting lattice parameters those of graphene and then relax them by total energy minimization; (2) we consider several starting positions of the G relative to the BP layer by shifting one another in finite amounts $\delta_{x}$ and $\delta_{y}$ (see Figure 2a,c); and (3) we optimized the interlayer distance.

After finding the equilibrium position of the phosphorene layer relative to the graphene one, we calculated the binding energy, per carbon atom, between the layer of phosphorene and graphene for different values of the interlayer distance *d* (see Figure 2d). The negative binding energy is given by

(1) $$-E_{b}=\left[E_{G/P}-\left(E_{G}+E_{P}\right)\right]⁄N$$

where $E_{G/P}$, $E_{G}$ and $E_{P}$ are the total energies of the G/P heterostructure, the freestanding graphene and the single layer of phosphorene, respectively, while *N* is the number of carbon atoms in the 4 × 13 supercell (N= 208). The equilibrium distance between the layer of graphene and the layer of phosphorene in the heterostructure G/P is about 3.5 Å, which is in good agreement with the theoretical results reported in the literature: interlayer distances between 3.45 and 3.76 Å are found if the vdW interaction is included depending on the details of the used vdW interaction treatment [37,38,53]. The calculated distance of 3.5 Å corresponds to the sum of the vdW radii 1.7 Å (C) and 1.8 Å (P) of the two forming atoms [54,55]. The value of our binding energy, 37 meV/C atom, is, however, somewhat smaller than the value from other vdW calculations [37,53] but still much larger than the energy without vdW interaction [56,57]. An important reason for the discrepancy is the almost absent strain in our studies using very large lateral unit cells and the modified stoichiometry compared with the smaller supercell case.

### 3.2. Band Structures and Schottky Barriers

**Figure 3:**
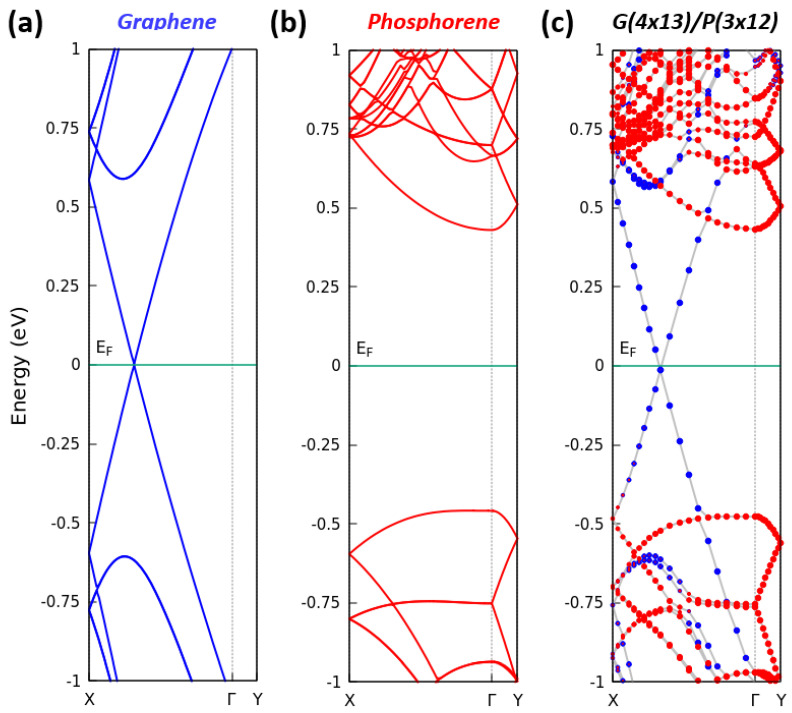
Calculated band structures of (**a**) pristine graphene, (**b**) puckered phosphorene and (**c**) G/P heterostructure. In (**c**), the colors of the circles correspond to the projection of the states on C (blue) or P (red) atoms. The Fermi level is set to zero.

In Figure 3, we report the band structures of the pristine graphene, pristine phosphorene and G/P heterostructure for the equilibrium interlayer distance equal to 3.5 Å. The band structures of graphene and phosphorene widely agree with the results of previous DFT calculations. Around the Fermi level that crosses the Dirac point, the cones in Figure 3a are characterized by linear bands $\hbar v_{F}\left|k\right|$ (being k in-plane Bloch vector) around the *K* or $K^{\prime }$ point in the BZ of the primitive hexagonal lattice, with a Fermi velocity of $v_{F}=8.3\times 10^{5}$ m/s, in agreement with other PBE studies [14]. The conduction and valence band extrema near ±0.7 eV are mainly due to the folding of electronic bands appearing in the primitive hexagonal BZ near the *M* points. Despite the use of a large 2D supercell, Figure 3b shows that puckered phosphorene is a direct semiconductor with a CBM and a VBM at Γ and has a DFT band gap of about 0.9 eV, in agreement with previous calculations based on the same methodology [4,13,58,59]. We know that the inclusion of many-body excitation aspects, e.g., within the GW approximation [60], increases both the Fermi velocity of graphene to a value which is slightly above $1\times 10^{6}$ m/s [14] and the quasi-particle gap of phosphorene toward 1.6, or even 1.83 eV [24,61]. The computation of quasi-particle corrections is only possible for systems with a few atoms in the unit cell. It is not feasible for systems with 208 (4 × 13 graphene), 144 (3 × 12 phosphorene) or 352 atoms in the G(4 × 13)/P(3 × 12) supercell of the heterostructure, mainly because of memory and CPU time requests.

**Figure 4:**
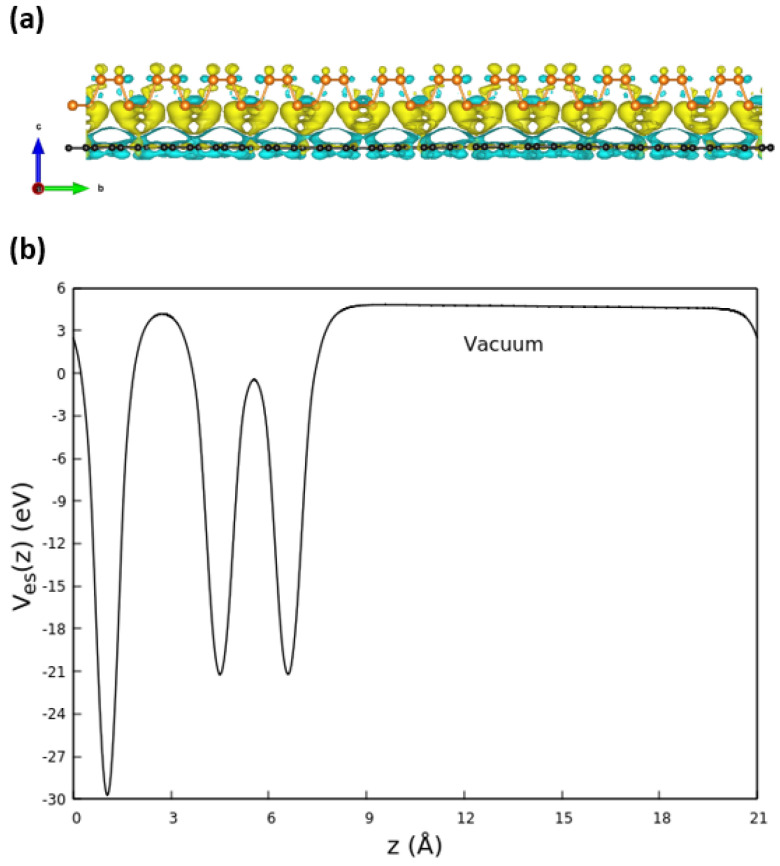
(**a**) Charge transfer between graphene and phosphorene: An isovalue of 5.95 × 10${}^{-5}$ a.u. is chosen for the electron density difference. Yellow color indicates the depletion of electrons, while cyan indicates the abundance of electrons. (**b**) Electrostatic potential averaged over the plane of the layers along one heterostructure and the vacuum region between two replicas.

The band structure of the combined G/P system in Figure 3c indicates only a minor influence of the vdW interaction between the two 2D crystals. The band structure of the heterostructure is basically given by the sum of the band structures of the two isolated layers. As part of the heterostructure, graphene has the same electronic properties of an isolated layer, showing the characteristic Dirac cone at *K* point. The typical phosphorene gap at Γ is larger in the heterostructure than in freestanding phosphorene. The distance between the phosphorene-derived CBM and VBM at Γ is somewhat increased from 0.9 eV to 1.06 eV. The reason for the weak influence of the mutual interaction between graphene and phosphorene is illustrated in Figure 4. There is only a weak charge transfer within the G(4 × 13)/P(3 × 12) heterostructure (see Figure 4a) for which electrons are transferred from phosphorene to the graphene layer. Despite the absence of inversion symmetry, Figure 4b shows the formation of a single extremely small dipole in the heterostructure, which modifies the plane-averaged electrostatic potential $V_{es}\left(z\right)$ by an internal electric field of $2.3\times 10^{6}$ V/cm in the vacuum region.

Utilizing the bilayer heterostructure, with the band structure given in Figure 3c, in an electronic or optoelectronic device, the graphene layer can be used as a metal contact, while the phosphorene layer may form the conducting channel. The device action of this metal–semiconductor contact is characterized within the Schottky–Mott model [62] by the Schottky barrier height (SBH) $\Phi_{B}$ for electrons (*n*-type) or holes (*p*-type) as distances of the CBM, $E_{C}$, or VBM, $E_{V}$, to the Fermi level $E_{F}$ according to

(2) $$\Phi_{Bn}=E_{C}-E_{F},$$

(3) $$\Phi_{Bp}=E_{F}-E_{V}.$$

For the G(4 × 13)/P(3 × 12) structure, we observe from the Kohn–Sham band structure in Figure 3c $\Phi_{Bn}=0.43$ eV and $\Phi_{Bp}=0.48$ eV. These values are nearly in agreement with the predictions by means of the band structures of the isolated 2D crystals in Figure 3a,b when applying the Fermi-level or the vacuum-level alignment [63]. Surprisingly, the Fermi-level alignment gives rather the same Schottky barriers as their direct calculation from Figure 3c. Moreover, the vacuum-level alignment technique seems to lead to reasonable predictions for the heterostructures with phosphorene [64] and has also been applied to the G/P system [37,44]. The energy distance between the vacuum level and the Fermi level gives the value of the work function *W* for a material. Comparing the work function $W_{G}=4.24$ eV of graphene with the $W_{P}=4.56$ eV of phosphorene results in a band bending on the order of 0.32 eV. Our equilibrium study indicates an *n*-type Schottky contact somewhat in contrast to the G(4 × 1)/P(3 × 1) strained heterostructures found in the literature [37,44]. Moreover, due to the use of hybrid functionals to generate approximate quasi-particle bands, the values found in the literature are anyway different, with $\Phi_{Bn}=0.66$ eV, $\Phi_{Bp}=0.34$ eV [44] or $\Phi_{Bn}=0.87$ eV, $\Phi_{Bp}=0.64$ eV [37] indicating a *p*-type Schottky contact. These results for graphene are opposite to conventional metals, which should give rise to *n*-type contacts [65].

### 3.3. Perturbations: Vertical Strain and Electric Field

A vertical tensile or compressive strain on the heterostructure leads to an increase or decrease in the interlayer distance *d*.

**Figure 5:**
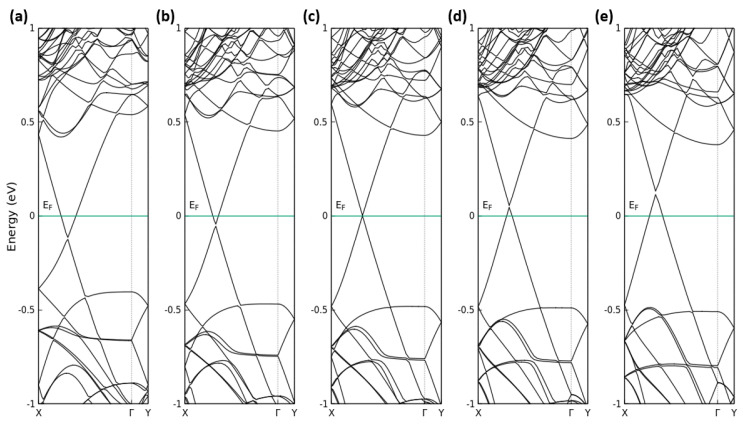
Calculated band structures of G(4 × 13)/P(3 × 12) heterostructure for different values of the interlayer distance: (**a**–**e**) *d* = 3.2 Å, 3.4 Å, 3.5 Å, 3.6 Å and 3.8 Å. The Fermi level is set to zero.

In Figure 5, we report the calculated band structures of G/P heterostructures for different values of the interlayer distance. Surprisingly, the layer distance has a significant influence on the electronic structure of the G(4 × 13)/P(3 × 12) heterostructures, mainly due to the modification/interaction of vertical C $2p_{z}$ and P $3p_{z}$ orbitals. For distances smaller than the equilibrium one d=3.5 Å, graphene (phosphorene)-derived conduction bands are lowered (shifted upward) but oppositely displaced for larger distances, while an opposite behavior is exhibited by the valence bands. Consequently, the CBM (VBM) becomes more graphene-like for smaller (larger) distances with band extrema outside Γ on the ΓX line. Going from a larger to a smaller value for *d*, the P-derived gap is only weakly decreased. The graphene-derived Dirac cones survive with a small gap of about 10 meV around the Dirac point. However, in Figure 5a, significant influence of the distance *d* on the position of the Dirac point is visible.

In fact, the Dirac cone moves below or above the Fermi level of the heterostructure and, in particular, when the interlayer distance is less than 3.5 Å, there is an *n*-type doping of graphene, while for distances greater than 3.5 Å, there is a *p*-type doping of graphene.

**Figure 6:**
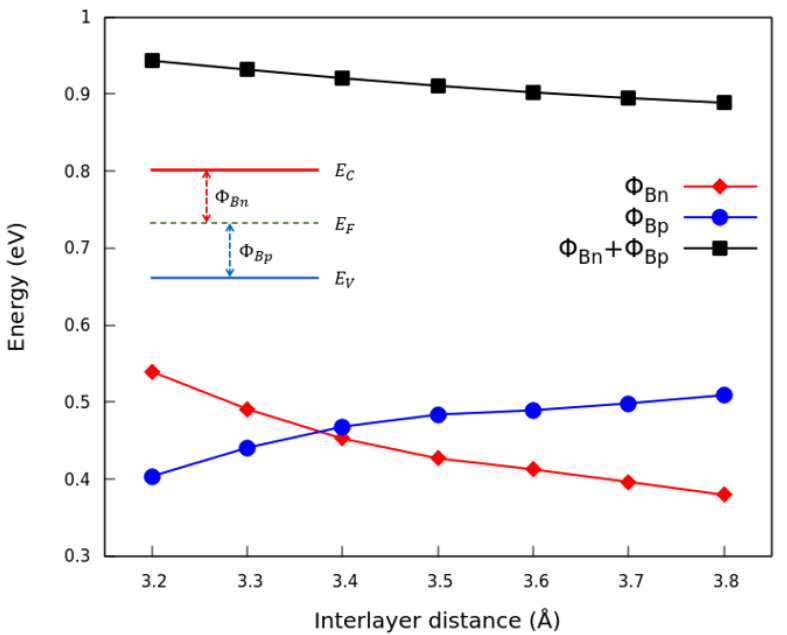
Schottky barriers $\Phi_{Bn}$, $\Phi_{Bp}$ and $\Phi_{Bn}$+$\Phi_{Bp}$ in the G(4 × 13)/P(3 × 12) heterostructure as a function of the interlayer distance.

The variation in the band edges and the Dirac point with the interlayer distance *d* has consequences for the Schottky barriers. From the graph shown in Figure 6, it is possible to see that for the interlayer distances above 3.4 Å, $\Phi_{Bp}$ becomes larger than $\Phi_{Bn}$, and there is a transition from a *p*-type Schottky contact to an *n*-type Schottky contact. The sum of the two barrier values $\Phi_{Bn}+\Phi_{Bp}$ shows a minor variation with the distance: it decreaseswhen the distance *d* rises, similar to the interband energy at Γ — the “gap”. The behavior of $\Phi_{Bn}$, $\Phi_{Bp}$ and $\Phi_{Bn}+\Phi_{Bp}$ is in qualitative agreement with another theoretical study [43].

The interaction between graphene and phosphorene in their heterostructure, but especially the resulting charge transfer, can be remarkably tuned by an external electric field *F* in the normal direction, i.e., perpendicular to the heterostructure.

**Figure 7:**
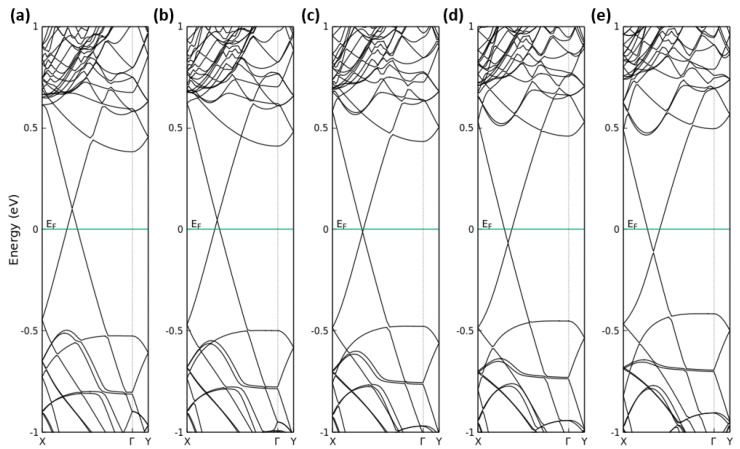
Calculated band structures of G(4 × 13)/P(3 × 12) heterostructure under an electric field: (**a**–**e**) *F* = −1.0 V/Å, −0.5 V/Å, 0.0 V/Å, 0.5 V/Å and 1.0 V/Å. The Fermi level is set to zero. The graphene–phosphorene distance *d* is the equilibrium one (3.5 Å).

**Figure 8:**
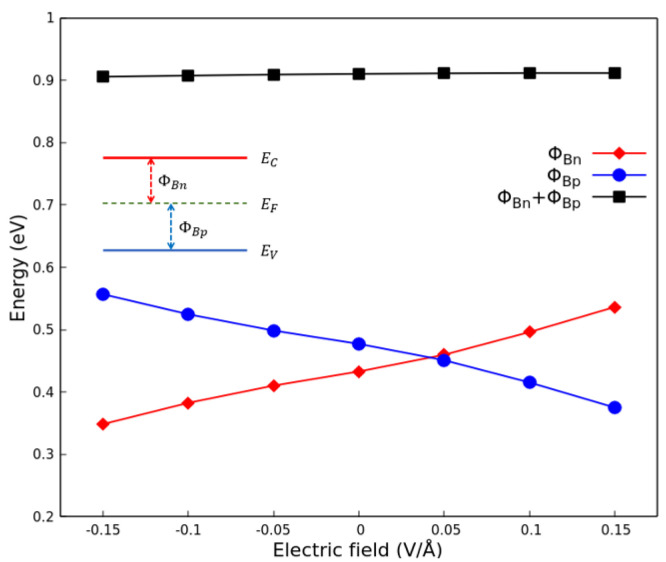
Schottky barriers $\Phi_{Bn}$, $\Phi_{Bp}$ and $\Phi_{Bn}$+$\Phi_{Bp}$ in the G(4 × 13)/P(3 × 12) heterostructure as a function of applied electric field. The graphene–phosphorene distance *d* is the equilibrium one (3.5 Å).

In Figure 7, we report the calculated band structures of the G(4 × 13)/P(3 × 12) heterostructure under different values of applied electric field; as visible, the graphene-related Dirac cone almost survives. Only the small gap of a few meV (approx. 7 meV) is somewhat opened toward large positive fields. More important is the movement of the Dirac cone away from the Fermi level. Negative (positive) fields move the Fermi level below (above) the Dirac point and, hence, give rise to a filling of the lower (upper) Dirac cone with holes (electrons). The band edges of the phosphorene-derived bands are hardly influenced so that the phosphorene-related interband distance, the “gap”, is almost conserved. The same holds for the energy distance between the extrema of the graphene-derived bands. The corresponding conduction band minimum and valence band maximum are displaced toward lower energy values with rising positive field strength *F*. For negative values of an applied electric field, we observe a gate-field-induced *p*-type doping of graphene. For positive values, instead, we obtain an *n*-type doping of graphene. Applying a positive electric field greater than 0.05 V/Å, the Schottky barrier $\Phi_{Bn}$ becomes larger than the Schottky barrier $\Phi_{Bp}$, leading to a transition from an *n*-type Schottky contact to a *p*-type Schottky contact, as shown in Figure 8. Both general findings are in line with predictions in the literature [37,44].

## 4. Summary and Conclusions

By means of density functional theory, we investigated the structural and electronic properties of graphene/phosphorene heterostructures under strain and an external vertical field. We focused especially on the resulting Schottky barrier between the two 2D crystals. To overcome the significantly large lateral strain of previous studies, caused by the employ of small unit cells, we investigated graphene (4 × 13)/phosphorene(3 × 12) coincidence lattices with very large supercells containing 352 atoms. We demonstrated that the electronic properties of graphene and phosphorene remain preserved upon their contact with an interaction within a typical vdW distance. We have also shown that varying the interlayer distance around the equilibrium values and the external electric field, it is possible to tune the positions of the band extrema in the electronic band structure of phosphorene relative to the Fermi level. Moreover, the Dirac cones of graphene surviving from the contact formation can be moved upward or downward by means of playing with the external perturbation. As a consequence, the upper band extrema will be filled with electrons, or holes will appear in the lower one. This behavior allows to control the Schottky barrier height of this metal–semiconductor bilayer system, enabling a switch in the conduction type of the Schottky contact. In fact, it changes from a *p*-type to an *n*-type Schottky contact from interlayer distances smaller to larger than 3.4 Å. Such a conductivity transition also occurs under the action of an external electric field for positive strengths bigger than 0.05 V/Å. In the equilibrium case (*d* = 3.5 Å and electric field strength F = 0), the less-strained heterostructure exhibits a Schottky barrier of opposite type compared with that of the significantly more strained systems studied in the literature. Summarizing, we demonstrated the possibility for the preparation of almost strain-free graphene/phosphorene contacts, which are fundamental for the design of novel elastic and anisotropic devices based on vdW heterostructures containing graphene as electrode and phosphorene as the material with the conducting channel. The range of values of the proposed G/P Schottky barriers compare well with those made up of gold and silicon. The advantages of using G/P for new devices are low cost, high flexibility and small size.

## Data Availability

The data presented in this study are available on request from the corresponding author.

## Abbreviations

The following abbreviations are used in this manuscript:

BP	Black Phosphorus
BZ	Brillouin Zone
CBM	Conduction Band Minimum
DFT	Density Functional Theory
FET	Field-Effect Transistor
G	Graphene
GGA	Generalized Gradient Approximation
G/P	Graphene/Phosphorene
P	Phosphorene
PBE	Perdew–Burke–Ernzerhof
SBH	Schottky Barrier Height
VBM	Valence Band Maximum
vdW	van der Waals
XC	eXchange Correlation
